# Fertility Sparing in Endometrial Cancer: Where Are We Now?

**DOI:** 10.3390/cancers17010112

**Published:** 2025-01-01

**Authors:** Gabriele Centini, Irene Colombi, Ilaria Ianes, Federica Perelli, Alessandro Ginetti, Alberto Cannoni, Nassir Habib, Ramon Rovira Negre, Francesco Giuseppe Martire, Diego Raimondo, Lucia Lazzeri, Errico Zupi

**Affiliations:** 1 Department of Molecular and Developmental Medicine, Obstetrics and Gynecological Clinic, University of Siena, 53100 Siena, Italy; irene.colombi@student.unisi.it (I.C.); i.ianes@student.unisi.it (I.I.); ginetti2@student.unisi.it (A.G.); alberto.cannoni@student.unisi.it (A.C.); francesco.martire@ao-siena.toscana.it (F.G.M.); lazzeri13@unisi.it (L.L.); errico.zupi@unisi.it (E.Z.); 2 Pediatric Gynecology Unit, Meyer Children’s Hospital IRCCS, 50139 Florence, Italy; federica.perelli@meyer.it; 3 Department of Obstetrics and Gynecology, Clinique de l’Yvette, 67 Route de Corbeil, 91160 Longjumeau, France; dr.nassirhabib@gmail.com; 4 Department of Gynecologic Oncology, Hospital de la Santa Creu i de Sant Pau, 08025 Barcelona, Spain; ramonroviran@gmail.com; 5 Division of Gynaecology and Human Reproduction Physiopathology, IRCCS Azienda Ospedaliero Univeristaria di Bologna, 40138 Bologna, Italy; diego.raimondo2@unibo.it

**Keywords:** endometrial cancer, fertility sparing, uterine neoplasms

## Abstract

Endometrial cancer is the most common gynecological neoplasm with an increased incidence in the premenopausal population in recent decades. This raises the problem of managing endometrial cancer in fertile women who have not yet achieved pregnancy. In these women, after careful selection, hysterectomy may be postponed in favor of conservative management if specific requirements are met. The latest evidence is focused on early endometrial carcinoma, endometrioid histotype, Grading 1, with no evidence of myometrial infiltration. Few clinical trials have opened this possibility also for women with an endometrial cancer Grading 2 diagnosis. There are still questions about the best medical therapy, dosage, route, and duration of treatment. Oral progestins or levonorgestrel-releasing intrauterine devices appear to be the options associated with the best outcome in terms of complete response and lower recurrence rates. Other options include the use of GnRH analogues, surgical hysteroscopy, or metformin, in a therapeutic approach that takes into account the characteristics of the patient. The pursuit of pregnancy should start as soon as two consecutive endometrial biopsies are obtained 3 months apart from each other; it is recommended to refer the patients to ART centers to maximize the success rate. After having reached the fulfillment of the reproductive desire, surgical radical treatment is still recommended.

## Simple Summary

Endometrial cancer no longer only concerns older women. The progressive increase in the age at first pregnancy requires individualized management of the disease in young patients who wish to preserve their fertility. Therefore, fertility-sparing treatment has become a topic of great interest. The aim of this manuscript is to summarize the existing literature on this topic in order to make it easily accessible to clinicians and to provide an overview of possible therapeutic strategies. We also aim to raise awareness of the issue among clinicians and encourage research. We believe that our manuscript will help in the management of young women with endometrial cancer who are planning to conceive, and we hope that it will stimulate new studies in this area.

## 1. Background

Endometrial cancer (EC) is currently the fourth most common cancer in women and the most common gynecological neoplasm [1]. Its incidence is increasing, and it is estimated that by 2030 it could be more common than colorectal cancer, becoming the fourth leading cause of cancer death in women [2]. The World Health Organization (WHO) estimates that there will be 130,051 cases of endometrial cancer in Europe in 2020, with 29,963 deaths [3].

Generally, it has a good prognosis. It is symptomatic from the onset of the disease, and it shows a 5-year survival rate of 81.1%, which increases to 94.9% for lesions confined to the uterine corpus [4].

It is well established in the literature that it is associated with several risk factors, including early menarche or late menopause, nulliparity, polycystic ovarian syndrome (PCOS), diabetes, obesity, tamoxifen therapy, pelvic radiation, and Lynch syndrome [5].

Diagnosis is anatomopathological, based on endometrial sampling. The workup of affected women is tailored to the hystotype and grading of the lesion, the extent of myometrial invasion, cervical infiltration, local pelvic spread, and the presence of distant metastases [6].

The diagnosis, classification, staging, and treatment of endometrial cancer are constantly evolving. In 1983, Bokhman [7] classified endometrial cancer into type I and type II. Type I, estrogen-dependent, accounts for about 85% of endometrial cancer cases and generally has a good prognosis. Type II, on the other hand, is rarer, estrogen-independent, and more often associated with a poor prognosis [8].

This division has now been overtaken by the introduction of four genomic categories proposed by The Cancer Genome Atlas [9] classification. These categories have recently been incorporated into the 2023 FIGO [10] classification, leading to a dramatic change in the staging of endometrial cancer. The ESGO-ESTRO-ESP group has also recently commented on the classification of endometrial cancer. One of the most interesting aspects is undoubtedly the stratification into prognostic risk groups according to the molecular signature, which has been shown to modify the stage of the disease in low-risk patients [6]. Definitive diagnosis and staging can only be achieved after hysterectomy, but preoperative staging based on a biopsy specimen and accurate imaging assessment is necessary to decide on the most appropriate management, as different approaches are required depending on the preoperative stage.

The first-line imaging techniques for preoperative staging are transvaginal ultrasound (TVS) and magnetic resonance imaging (MRI). TVS shows some advantages: it is cheaper than MRI and available worldwide yet has comparable diagnostic accuracy for preoperative assessment of myometrial and cervical invasion in endometrial cancer [11], with a sensitivity of 75% and a specificity of 82% [12]. A consensus on nomenclature has been reached in accordance with the International Endometrial Tumour Analysis (IETA) [13] to ensure standardized observation and description. Several ultrasonographic characteristics have been associated with the presence of endometrial cancer: the ipoechogenicity of the endometrium with inhomogeneous aspects, the absence of a visible midline or bright edge, and high vascularity with irregular multiple vessels of multifocal origin. The crucial phase is the assessment of myometrial and cervical infiltration, as this is an important factor in establishing the pre-surgical stage of the disease. The aim is to define whether or not the endometrium affected is infiltrating the myometrium and, if so, to assess its extent by dividing the patients into cases in which the myometrium is infiltrated for <50% of its thickness or for >50%. This initial assessment allows us to determine whether the disease is at stage IA or IB according to the 2023 FIGO classification [10]. If the infiltration is full thickness and reaches the uterine serosa, the patient is classified as stage IIIA. Some lesions may infiltrate the cervix, which must be carefully analyzed, as its involvement further modifies the stage and places the patient at stage II.

Although endometrial cancer is a disease whose prevalence correlates with increasing age, with an average age of 63 years, its incidence in the premenopausal population has increased in recent decades. More specifically, its incidence is 15–25% in women of reproductive age [14], of which 4–10% are diagnosed in under-40-year-olds [15,16].

The concomitant progressive increase in the average age at first pregnancy raises the problem of preserving the patient’s reproductive potential. Treatment strategies must strike a balance between allowing pregnancy to occur and limiting the risks of disease progression and recurrence [14].

This alarming scenario highlights the global health impact of endometrial cancer and the crucial urgency of effective interventions, especially for women who wish to preserve fertility.

Fortunately, the endometrial lesions found in young patients are most often focal, confined to the endometrium with only superficial myometrial invasion, endometrioid histotype, Grade 1, and well differentiated and are therefore generally associated with a good prognosis, allowing for the possibility of conservative treatments [16].

In 2023, the European Society of Gynaecological Oncology (ESGO), the European Society of Human Reproduction (ESHRE), and the European Society of Gynaecological Endoscopy (ESGE) provided new guidelines for fertility-sparing treatment in women with endometrial cancer [17].

The present narrative review aims to provide an update of the last 10 years of literature on fertility-sparing indications, strategies, and outcomes for women with endometrial cancer who wish to preserve fertility.

## 2. Materials and Methods

This manuscript is a narrative review. We searched PubMed for relevant articles from January 2014 to July 2024. We used the following search string: “fertility sparing AND endometrial cancer”. We selected only articles on women with endometrial cancer who were eligible for fertility-sparing treatment, written in English, and published within the time period relevant to the research question.

Studies that did not meet the established inclusion criteria, duplicate studies, non-peer-reviewed papers, grey literature, or reports with insufficient scientific rigor were excluded.

We retrieved 351 papers, 4 of which were not considered because the full text was not available. After removing meta-analyses, reviews, and systematic reviews, a total of 6 publications were included in our manuscript. Two independent reviewers (I.C. and I.I.) assessed all titles and abstracts; 1 trial was finally eliminated because it did not focus on the topic of the current review, and 1 paper was excluded because it was not written in English.

The process conformed to the recommendations of the Preferred Reporting Items for Systematic Reviews and Meta-Analyses (PRISMA) [18]. The protocol was not registered.

**Figure 1:**
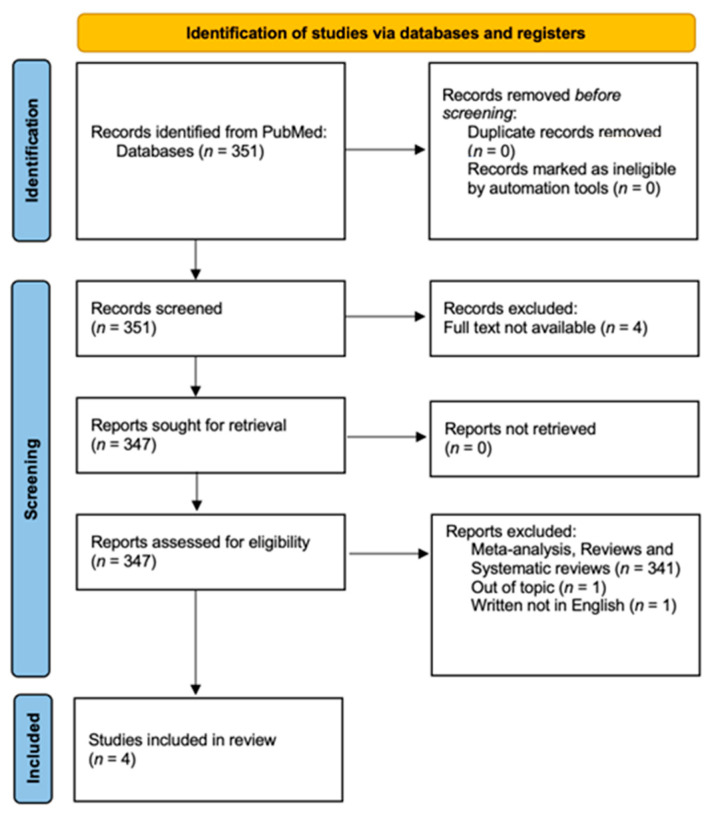
Literature search diagram, PRISMA [18] 2020 flow diagram, which includes searches of PubMed. A total of 351 papers filled the search string. Of these, 4 articles were excluded because the full text was not available. In addition, 341 were excluded because they were meta-analyses, reviews, or systematic reviews; only clinical trials and controlled trials were included. A total of 6 papers were eligible for review. After evaluating the titles and abstracts, 1 article was excluded because it was not relevant to the topic of the review, and 1 article was excluded because it was not written in English.

Figure 1 shows the flowchart of the literature search. Four articles were identified for review (Table 1).

To provide an appropriate background and description of the state of the art in fertility-sparing treatment of endometrial cancer, a further electronic search was conducted using the online medical database MEDLINE (accessed via PubMed) to examine the published literature on this topic. Two independent reviewers (I.C. and I.I.) selected articles relevant to our research question. 

Finally, all included articles were carefully assessed for their scientific merit and relevancy.

## 3. Results

### 3.1. Eligibility for Fertility-Sparing Treatment

In carefully selected patients who wish to preserve reproductive function, hysterectomy can be postponed [23].

According to the 2023 ESGO/ESHRE/ESGE guidelines [17], patients who meet the following criteria are considered eligible for conservative treatment:

- Biopsy diagnosis of endometrial hyperplasia with atypia;
- Biopsy diagnosis of intraepithelial neoplasia (EIN);
- Biopsy diagnosis of endometrial carcinoma, endometrioid histotype, Grading 1;
- No evidence of myometrial infiltration;
- No evidence of extrauterine pathology on imaging (TVS/RM/CT);
- Reproductive desire with immediate pregnancy plan.

In the case of hyperplasia without atypia, the long-term risk for progression to endometrial carcinoma is less than 5% [24]. This justifies the option of watchful waiting as management, having a spontaneous regression rate of 70 to 100%; alternatively, medical treatment options can be considered without the need for demolitive surgery [24].

To date, it is unclear whether EC in young patients with a mismatch repair (MMR) mutation or a BReast CAncer Gene 1–2 (BRCA1–2) mutation are eligible or not for fertility-sparing treatment. Some authors suggest that conservative treatment could be offered to this subgroup of patients after appropriate counselling, as conservative treatment is seen as a limited window of opportunity for conception, followed by definitive surgery [25].

Since pregnancy is the primary goal of conservative management, an assessment of the woman’s reproductive potential should be made before proceeding with fertility-sparing treatment. To evaluate the patients’ eligibility for fertility-sparing approach, it is important to carry out an accurate assessment of the clinical history and reproductive potential of the woman and the couple. Women’s childbearing potential is examined by ovarian reserve, measuring Anti Mullerian Hormone (AMH), the Antral follicle count (AFC), and the Follicle Stimulating Hormone (FSH) values on days 2 and 5 of the menstrual cycle. The patient’s age and weight must be carefully considered, as they are closely associated with ovarian reserve and function and the success rate of controlled stimulation cycles during medically Assisted Reproductive Technology (ART) [17]. In multiple literature studies, reproductive possibilities were greater in patients under 35 years old, with a live birth rate of 30.7% and a progressive decrease as the patient’s age increased. Considering patients up to 40 years old, the rate of live birth is around 23% [26]. Regarding women’s body weight, a high Body Mass Index (BMI) has a negative impact on reproduction and is associated with infertility, increased time-to-pregnancy, and worse pregnancy outcomes [27]. Therefore, the maintenance of adequate body weight, a healthy lifestyle, and bariatric surgery in obese patients are all factors associated with a reduction in the risk of endometrial cancer [17] and an improvement in reproductive chances [28]. Zhang et al. [24] demonstrated how a weight loss > 5% significantly increases the reproductive chances and the live birth rate. Women should also be encouraged to maintain a BMI < 30 kg/m^2^, as neoplastic relapse is much more common in obese patients.

We should also not underestimate the fact that neoplasms are associated with psychological and social stress or mental disorders, so psychological assessment may need to be considered in these patients prior to treatment.

After ensuring that the reproductive potential of the patient and couple is adequate, the anatomopathological features of endometrial cancer should be considered to select patients who can be enrolled in conservative management strategies. In the event of endometrial carcinoma, only patients with endometrioid histotype, G1, stage IA (FIGO), without myometrial invasion are considered candidates for fertility-sparing treatment. Patients with grade 1 endometrioid carcinomas, hormone receptor positivity, small focal lesions < 2 cm, in the absence of myometrial, adnexal, and lymphovascular space involvement, show an excellent prognosis, with a 5-year survival rate of 95%.

The debate in the literature remains open, however, in the case of Grading 2 lesions. Few studies have investigated the possibility of offering conservative treatment in this subgroup of patients. Hwang et al. [29] reported a complete response to treatment with Medroxyprogesterone acetate (MPA) and simultaneous placement of a levonorgestrel medicated IUD in three out of five patients with G2 stage IA endometrial cancer over 11 months of therapy. Superimposed data emerged from the work of Shan et al. [30] following the administration of 1500 mg of metformin and 160 mg of megestrol acetate each per day. This management obtained a 75% complete regression of the pathology rate.

The largest series of patients diagnosed with intramucosal, G2, endometrioid EC who were selected for fertility-sparing treatment were 23 women, treated by progestin therapy (with or without hormonal replacement therapy). After a median follow-up of 3 years, 73.9% of them achieved complete response, with a recurrence rate of 41.1%. Almost all the recurrences were treated with definitive surgery, while one patient declined the surgery and went for conservative re-treatment, obtaining again a complete response. The results show also a longer time to response to progestin in G2 EC compared to the G1, at 6 months vs. 4.5 months [25].

Also, in the study of Yu et al. [31] on eight patients with stage IA G2 endometrial cancer, the response was complete in seven patients (87.5%). The presence of estrogen and progesterone receptors seems to have a favorable prognostic role [17]; in particular, the presence of progesterone receptors is associated with higher rates of complete resolution after treatment with medroxyprogesterone acetate [32]. The best predictor of response to hormonal therapy remains the degree of differentiation of the lesion [32].

### 3.2. Treatment Strategies

#### 3.2.1. Progestins

Conservative therapy is based on the use of progestins in oral formulations or levonorgestrel-releasing intrauterine devices [23]. The response to progestins is satisfactory in 76–81% of cases with differences between the formulations.

In a meta-analysis of various fertility-sparing therapies involving 661 patients in 38 trials, the oral progestin megestrol acetate, 160–320 mg daily, was associated with the highest remission rate, while medroxyprogesterone acetate, 400–600 mg daily, was associated with a lower risk of recurrence [4,33].

Casadio et al. [16] observed complete remission of the disease in 94.4% of patients in a cohort of 36 women with endometrial cancer, treated with 160 mg megestrol acetate; only in two cases was the lesion still present at the first control after the beginning of treatment.

The response to oral progestogen treatments was complete in 65.8% of women with endometrial hyperplasia with atypia, which were 55 in total in the study, and in 48.2% of women with endometrial cancer. The recurrence rates, however, remained at 23.2% and 35.4%, respectively.

A possible alternative to oral treatment is the placement of a levonorgestrel-releasing intrauterine device (LNG-IUD), which is associated with reduced systemic side effects and better compliance by the patient [4].

**Figure 2:**
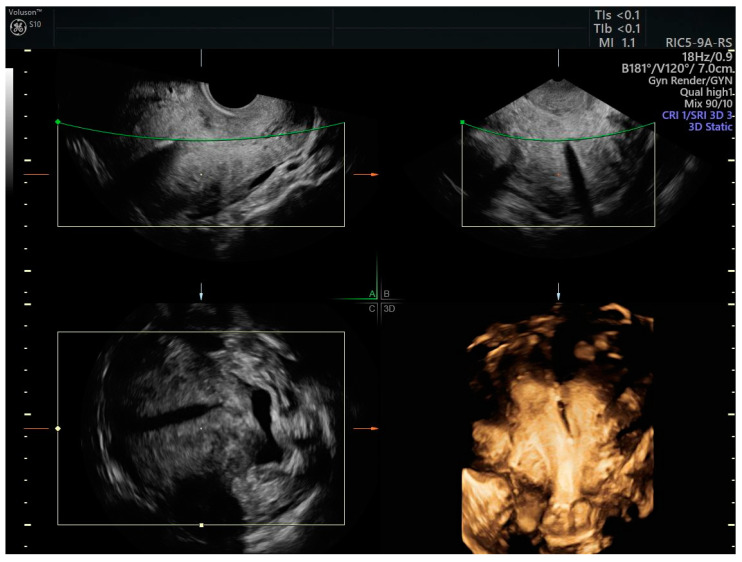
3D scan of the uterus of a patient with endometrial cancer treated with LNG-IUD. The correct positioning of the intrauterine device is monitored by ultrasound, which also allows the identification of any changes in the endometrial pattern during treatment.

The LNG-IUD (Figure 2) in 57 patients (21 endometrial cancer, 36 complex atypical hyperplasia) showed a complete response in 91% of women with hyperplasia with atypia and 54% of women with endometrial cancer within 12 months of treatment [23].

The two routes of administration (oral and intrauterine) can also be used simultaneously with satisfactory results in terms of reduction in recurrence rate compared to the use of LNG-IUD in monotherapy [34,35].

A risk factor for failure of progestin therapy is obesity (BMI ≥ 30), which is associated with a lower probability of achieving a complete response and an increased risk of recurrence in the long term. The reason is more likely to be found in an excess of endogenous estrogens that consistently antagonize progestin and decrease its therapeutic effects. For this reason, weight reduction is strongly recommended for obese patients during treatment and follow-up [36].

#### 3.2.2. Other Strategies

Other possible treatment strategies include GnRH analogues, either alone or in combination with LNG-IUD or aromatase inhibitors. This approach has been proven particularly suitable for the treatment of obese patients in which progestogen alone is often less effective [37].

**Figure 3:**
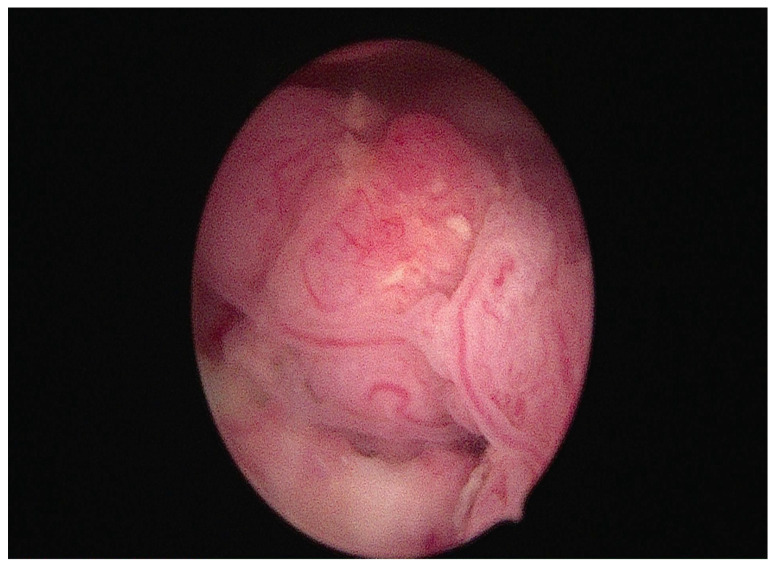
Hysteroscopic view of endometrial cancer with exophytic growth within the uterine cavity, treated with a tissue removal device.

Among the new treatment options, some authors suggest performing an operative hysteroscopy with a pre-treated endometrium resection to promote complete cytoreduction before progestin therapy [16,35,38]. Figure 3 shows a hysteroscopic image of a neoplastic endometrial lesion occupying the uterine cavity, treated with a tissue removal device.

**Figure 4:**
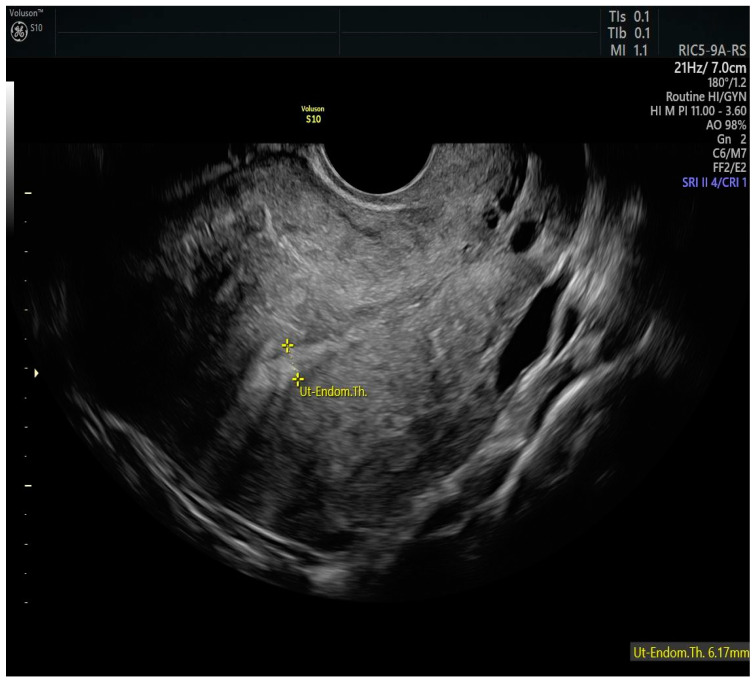
Two-dimensional longitudinal scan of the uterus of a patient treated with a levorgestrel-releasing IUD as part of a fertility-sparing regimen. The intrauterine device allows the thickness of the endometrium to remain reduced.

In 2005, Mazzon [39] described the method for the resection of endometrial carcinoma in three steps. In the first surgical step, the neoplastic lesion is resected; in the second step, 4–5 mm of the endometrium adjacent to the lesion is removed; and in the third step, 3–4 mm of the myometrium is removed below the previously removed neoplastic lesion. If confirmed by the pathological examination of G1, the patient can be referred to medical therapy with megestrol acetate 160 mg per day for 6 months. Giampaolino et al. [40] proposed to add additional random endometrial biopsies to the Mazzon technique and replace treatment with megestrol acetate by LNG-IUD placement. Figure 4 shows the case of a woman conservatively treated for endometrial cancer with LNG-IUD, with a thin endometrium. 

Hysteroscopic treatment followed by oral or intrauterine progestogen showed complete response in 90–95.3% of cases versus 76.3–77.7% during oral progestogen therapy alone and 71.2–72.9% during combined treatment with oral progestogen and LNG-IUD [17].

Hysteroscopic pretreatment also seems to offer advantages in terms of live birth rate [26]. Several meta-analyses reported a live birth rate of 53% in patients pretreated with operative hysteroscopy versus 33% in those treated only with progestins [41].

Laurelli et al. [21] demonstrate a durable complete response in 81% of the patients treated with the three-step tumor resectoscopy and the subsequent LNG-IUD insertion, with a median duration of 88 months.

All the patients were diagnosed with EC with progesterone receptors (PRs) at 50% or greater positivity at immunohistochemistry. The pregnancy and live birth rates were respectively 92% and 83% among the patients who attempted to conceive. This finding also suggests that the addition of hysteroscopic resection does not affect reproductive outcomes if performed with a standardized technique. The authors emphasize the importance of an accurate pre-treatment patient selection to achieve the best result in terms of conception [21].

Endometrium ablation is also a safe option but is not the first-line treatment. The limitations of this technique are the difficulty in confirming the complete ablation of the endometrium and the difficulty in the hysteroscopic follow-ups due to the subsequent obliteration of the endometrial cavity [24].

It has been previously hypothesized that increased intrauterine pressures promoted by distending media used in hysteroscopic procedures could contribute to the circulation of malignant cells in the peritoneal cavity through the fallopian tubes. Therefore, it can be assumed that hysteroscopic morcellation can further contribute to retrograde dissemination into the peritoneal cavity and adjacent tissue due to the high-pressure system. This concern was not confirmed in a retrospective work that involved 289 patients, demonstrating that hysteroscopic morcellation does not lead to increased dissemination of malignant endometrial cells in comparison with alternative methods of biopsy [42].

It has also been a topic of study to examine the possibility of conservative re-treatment of later recurrence of atypical endometrial hyperplasia and early endometrial cancer. Raffone et al. [43] conservatively re-treated 190 patients using oral progestin; a complete response was reported in 85.3% of them, with a subsequent disease recurrence of 40.4%. There is certainly a need to better investigate the rates of recurrence of AEH and EC separately and the treatment modality, but it is promising to potentially consider re-treatment as a safe and viable option in women who wish to attempt childbearing.

Among young women with endometrial cancer, 84% are obese, 83% have insulin resistance, and 78% have abnormal glucose metabolism [19]. All these mechanisms play a role in the increased incidence of cancer.

Metformin seems to reduce the incidence of cancer in diabetic patients and inhibits the growth of several neoplasms, such as breast, ovarian, and endometrial; it increases ovulation in patients with polycystic ovary syndrome and improves pregnancy rates [19]. Metformin has a role in altering glucose metabolism and inhibiting the PI3K-AKT-mTOR signaling pathway. It was also shown to increase progesterone receptor expression and sensitize progestin-resistant endometrial cancer cells to medroxyprogesterone-induced apoptosis. It was demonstrated how using metformin (500 mg, orally, three times a day) in addition to megestrolacetate increased the rates of reversing atypical endometrial hyperplasia to normal endometrial histology. However, its role has yet to be studied in endometrial cancer [20].

Based on the same pathway, its role was also evaluated in preventing recurrence after MPA treatment. When using metformin at the maximum dose of 2250 mg/die (if no adverse effects occurred) after achieving complete response with MPA treatment, only 10.3% of the patients relapsed during a median follow-up period of 38 months after remission. This protocol demonstrated that metformin inhibited EC recurrence and prolonged relapse-free survival after MPA therapy, bearing in mind the recurrence rates without metformin were 40.6% [22].

#### 3.2.3. Duration of Treatment

Although the best length of treatment has not yet been established, several authors believe that regression of the pathology occurs in 4–6 months after the beginning of treatment in most patients; this time can be longer in the case of obesity or insulin resistance [17]. The recommended treatment involves a therapy of 6–12 months, possibly prolonged to a maximum of 15.

Another meta-analysis of 24 studies involving 370 patients undergoing fertility-sparing treatment showed that the remission probability after 6 months of treatment was 72% compared to 78% after 12 months of treatment, suggesting marginal benefits beyond the first 6 months. Based on these data, non-responders at the 12-month follow-up with persistent disease confirmed on biopsies should be counseled to undergo hysterectomy [44].

According to the European Society of Gynecological Oncology recommendation, it should be considered unsuitable for patients who show persistent or progressive disease after 6 months of progestin treatment to continue with conservative management [31].

The factors involved in a reduced response to progestogen treatment are still not well defined, but it is hypothesized that factors include a role in the molecular pattern together with BMI > 25, high HE-4 values, low histological degrees, and polycystic appearance of the ovaries at TVS [17]. The diagnosis of PCOS is matched with obesity, insulin resistance, and chronic anovulation, which are risk factors for premenopausal EC and/or AEH and also associated with a poorer response to conservative treatment compared to non-PCOS patients [45].

### 3.3. Follow Up and Pre-Conception Counselling

There is no consensus across gynecologic oncology societies regarding the frequency of follow-ups and method of endometrial sampling. Strict observation is mandatory, as no standardized time frame for follow-up has yet been established. Most authors agree that patients should have an endometrial biopsy after 3 months and 6 months from the beginning of treatment [16], with simultaneous ultrasound evaluation.

The primary objective of conservative treatment is to achieve pregnancy. Therefore, when two consecutive biopsies are obtained 3 months apart from each other and the endometrial pathology is negative, the patient can try to conceive. To optimize the time to pregnancy and improve the reproductive chances, it is recommended to refer the patients to ART centers. Until a pregnancy test is positive, regular monitoring with biopsies every 3–6 months is recommended, or until definitive surgical treatment if reproductive desire is exhausted [17]. Other authors suggest follow-up with hysteroscopic biopsies every 3 months for 1 year and every 6 months for the next 4 years in all patients who have no immediate reproductive desire or after childbirth in patients who wish to have another pregnancy [16].

The decision to proceed with definitive treatment in the event of a negative follow-up after the end of reproductive desire is an individual one, and to date, there is no consensus on the timing of a possible hysterectomy; it may even be possible to continue with follow-up without considering definitive surgery [16]. In patients who refuse definitive treatment, progestogen therapy should be continued by maintaining the LNG IUD [17]. Oncological follow-up requires regular check-ups and strict adherence to the prescribed treatment, so patients must be adequately informed about the modalities of follow-up to ensure optimal compliance. In the case of indication for surgery, ovarian preservation is an option to be considered in premenopausal patients after careful evaluation of the patient’s age, familiarity with neoplasms, genetic conditions, or lesions found on the attachments [17].

The disease-free survival rate appears to be improved in the cohort of women who became pregnant than in those who did not. This indicates that achieving pregnancy may be associated with disease-free survival. In general, the mortality related to conservative treatment of EC is low and still comparable with that of primary hysterectomy [44].

The importance of proper informed consent and strict follow-up procedures for patients who choose fertility-sparing treatment for EC is fundamental. Obesity, polycystic ovarian syndrome, and anovulation are all conditions that predispose one to infertility; these patients should be encouraged to seek ART for conception as soon as a complete response is achieved [44].

To maximize the possibility of live births and minimize the time between diagnosis and definitive treatment, it is recommended that patients who achieve a complete response consult a reproductive specialist [31].

Pregnancy appears to be a protective factor against relapse and is associated with a lower risk of recurrence. Therefore, responders should be encouraged to seek referral for assisted reproductive technology, especially those with unfavorable pregnancy risk factors [36].

### 3.4. The Role of Molecular Patterns in Fertility-Sparing Treatment

More than a decade ago, The Cancer Genome Atlas [9] classified endometrial cancer into four genomic categories: POLE ultramutated (POLEmut), microsatellite instability hypermutated or mismatch repair deficient (MSI-H or MMRd), low somatic copy number alteration or non-specific molecular profiles (CNL or NSMP), and high somatic copy number alteration or p53 abnormal (CNH or p53abn). Prognosis varies between categories, with POLEmut generally better and p53abn worse.

Further studies validated this classification. The major contribution to this process of validation comes from the ProMisE study (i.e., Proactive Molecular Risk Classifier for Endometrial Cancer) [46].

The authors aimed to provide simplified tools for the assessment of endometrial cancer that can be used in most anatomic pathology laboratories and that would allow the introduction of molecular classification into clinical practice.

This methodology involves the gene sequencing of POLE and the use of immunohistochemical surrogates analogous to the known genomic subcategories of endometrial cancer established by the TCGA.

The four categories do not generally overlap, although in 3% of cases more than one molecular signature can be detected, defining the so-called multiple classifiers [47].

In 2023, FIGO [10] implemented molecular classification in the new staging system for endometrial cancer and proposed a categorization into subgroups to indicate more appropriate surgical, radiotherapy, or systemic therapies. Different molecular patterns were found to be associated with different recurrence risks and survival rates. Based on this new implementation, patients are categorized as low, intermediate, high intermediate, and high risk, and the stage may change according to the p53 or POLE status in stages I and II, determining an upstaging or downstaging of the disease (IICm p53abn or IAmPOLEmut) [6].

The evaluation of the molecular profile of the lesions could play a role in the future selection of patients eligible for conservative treatment. Some mutations are associated with a better prognosis than others. From the work of Tanos et al. [48], it was found that PTEN and POLE mutations are associated with better outcomes and better results after fertility-sparing treatment; conversely, the positivity of MSI mutations, CTNNB1, and K-RAS is associated with increased risk of recurrence while not excluding the possibility of conservative treatments. For PIK3CA, HER2, ARID1A, P53, L1CAM, and FGFR2, the prognosis is more often unfavorable, and the possibilities of conservative treatment require a case-by-case evaluation given the high risk of recurrence [14]. Notably, L1-CAM is an independent risk factor associated with both locoregional and distant dissemination, and CTNNB1 is associated with a higher risk of recurrence, requiring further studies to establish their role in the eligibility of fertility-sparing treatment. However, the literature is not yet sufficient to recommend routine molecular classification in clinical practice.

Falcone et al. [49] carried out a study trying to incorporate the ProMisE classification in the assessment of eligibility for fertility-sparing treatment, to better stratify recurrence risk and to evaluate the genetic risk of the individual patient. The results showed that approximately 50% of the patients had MMR defects, mutations that correlated with clinical outcome showing EC persistence/progression or metachronous Lynch syndrome-associated tumors. In contrast, only two of the eight patients with no mutations or POLE mutations showed unfavorable events. This is just one of the first applications of the ProMisE classification, which seems promising and potentially useful; genetic high-risk patients could be identified earlier and further counseled about their prognosis with conservative management [49].

## 4. Conclusions

The standard of care for endometrial cancer has changed dramatically in recent years [50], so it is vital to keep up with the latest advances and provide the best and most up-to-date treatment for this evolving disease. Fertility-sparing treatment is a valid option that should be offered to young patients with reproductive desire who meet the eligibility criteria after adequate evaluation based on endometrial biopsy [51], imaging assessment of the disease, and clinical and reproductive history. It can also be considered a valid option for patients with low-risk malignancies who refuse surgery or who are not candidates due to unfavorable clinical conditions [52]. The implementation of this conservative approach in clinical practice makes it possible to reduce the number of hysterectomies and, particularly in view of the increasing incidence of endometrial cancer in young women and the delay in pregnancy, allows more women to fulfil their reproductive desires.

## Figures and Tables

**Table 1:** Summary table of the main characteristics of the studies included in the review.

	Nation	Primary EndPoint	Secondary Endpoints	Inclusion Criteria	Study Design	Results
Mitsuhashi et al. BMJ Open. 2020 [19]	Japan, 2019	3-year relapse-free survival (RFS) rate	- RFS rate - MPA treatment overall response rate - Post-treatment conception rate - Pregnancy outcome - Assessment of toxicity and changes in insulin resistance and body mass index (BMI)	- Histologically documented AEH or endometrioid adenocarcinoma - Stage IA, confined to the endometrium - Not previously treated - Over 20 years old and under 42 years old	Three treatment arms for 32 weeks A: 600 mg MPA alone; B: MPA + metformin 750 mg/day; C: MPA + metformin 1500 mg/day Women underwent endometrial curettage every 8 weeks At the end of treatment, endometrial sampling every 3 months for 3 years	- MPA treatment response rate of 80% - 5% withdrawal probability during the maintenance period - 10% chance of pregnancy
Yang BY et al. BJOG. 2020 [20]	China, 2020	complete response (CR) rates within 16 weeks of treatment (16 w-CR rate)	30 w-CR rate	- Pathological diagnoses with AEH or EEC (endometrioid endometrial cancer, grade I, without myometrial invasion) limited to the endometrium - desire to preserve fertility - had no contraindication for metformin, megestrol acetate (MA), or pregnancy - had no hormone or metformin treatment within six months before entering the trial - were not pregnant when participating in the trial - Over 18 years of age and under 45 years of age	- ARM A: 160 mg MA alone - ARM B: MA + metformin 1500 mg/day Duration: 30 weeks Hysteroscopic evaluation every 3 months (removal of suspicious lesion + random biopsy) Follow-up: endometrial biopsy by Pipelle and TVUS every 3–6 months Enhanced pelvic MR, serum CA-125, and serum HE4 annually	- 39.6% (vs. 20,4%) 16 w-CR rate in AEH patients during the MA + metformin treatment - 48.4% pregnancy rates in arm A, 51.8% in arm B
Laurelli G et al. Int J Gynecol Cancer. 2016 [21]	Italy, 2016	long-term oncologic and reproductive outcomes	NA	- Pathological diagnosis of well-differentiated (G1) endometrioid endometrial cancer (EEC) with PR > 50% positivity at immunohistochemistry limited to the endometrium - Normal CA125 serum levels - No contraindication for progestin treatment - Over 18 years of age and under 45 years of age	Hysteroscopic resection (HR) to resect the tumor lesion, the endometrium adjacent and the myometrium underlying the tumor + Levonorgestrel intrauterine device (LNG-IUD) 52 mg. Duration: 6 months Follow-up: TVUS, serum CA125 and diagnostic hysteroscopy every 3 months, abdomen CT every 6 months.	- 85.7% complete regression, 9.5% persistent disease, 4.8% progressive disease - 10.5% recurrences - in the responders’ group, 92% pregnancy rate and 83% live birth rate
Mitsuhashi A et al. Ann Oncol. 2016 [22]	Japan, 2016	Relapse-free survival (RFS) after remission	- the efficacy of MPA with metformin, including overall response - the safety of and degree of toxicity with this combination - the changes in metabolic status, including in insulin resistance and BMI - the conception rate following treatment	- Histologically confirmed AEH or well-differentiated adenocarcinoma grade 1, limited to the endometrium - Over 20 years of age and under 40 years of age	MPA 400 mg/die + 100 mg Aspirin + Metformin (initial dose: 750 mg/die, increased weekly by 750 mg up to 2250 mg/die if no adverse effects occurred). Metformin therapy was continued after MPA administration until conception or disease recurrence. Duration: 24 weeks. Follow-up: median 38 months (range, 9–66 months)	- 64% CR within 6 months, 17% achieved CR within 9 months - 81% remission - 89.7 RFS, 10.3% relapsed during a median follow-up period of 38 months

## Data Availability

The data presented in this study are available on request from the corresponding author.
